# Stackelberg Evolutionary Games of Cancer Treatment: What Treatment Strategy to Choose if Cancer Can be Stabilized?

**DOI:** 10.1007/s13235-024-00609-z

**Published:** 2024-12-14

**Authors:** Monica Salvioli, Hasti Garjani, Mohammadreza Satouri, Mark Broom, Yannick Viossat, Joel S. Brown, Johan Dubbeldam, Kateřina Staňková

**Affiliations:** 1 Evolutionary Game Theory Lab, Faculty of Technology, Policy and Management, Delft University of Technology, Delft, The Netherlands; 2 Delft Institute of Applied Mathematics, Delft University of Technology, Delft, The Netherlands; 3 Department of Mathematics, City, University of London, London, UK; 4 Ceremade, Université Paris Dauphine-PSL, Paris, France; 5 Integrated Mathematical Oncology, H. Lee Moffitt Cancer and Research Institute, Tampa, FL USA; 6 Department of Advanced Computing Sciences, Maastricht University, Maastricht, The Netherlands

**Keywords:** Stackelberg evolutionary games, Evolutionary cancer therapy, Evolutionary game theory, Resistance, Heterogeneity, Mathematical oncology

## Abstract

We present a game-theoretic model of a polymorphic cancer cell population where the treatment-induced resistance is a quantitative evolving trait. When stabilization of the tumor burden is possible, we expand the model into a Stackelberg evolutionary game, where the physician is the leader and the cancer cells are followers. The physician chooses a treatment dose to maximize an objective function that is a proxy of the patient’s quality of life. In response, the cancer cells evolve a resistance level that maximizes their proliferation and survival. Assuming that cancer is in its ecological equilibrium, we compare the outcomes of three different treatment strategies: giving the maximum tolerable dose throughout, corresponding to the standard of care for most metastatic cancers, an ecologically enlightened therapy, where the physician anticipates the short-run, ecological response of cancer cells to their treatment, but not the evolution of resistance to treatment, and an evolutionarily enlightened therapy, where the physician anticipates both ecological and evolutionary consequences of the treatment. Of the three therapeutic strategies, the evolutionarily enlightened therapy leads to the highest values of the objective function, the lowest treatment dose, and the lowest treatment-induced resistance. Conversely, in our model, the maximum tolerable dose leads to the worst values of the objective function, the highest treatment dose, and the highest treatment-induced resistance.

## Introduction

Metastatic cancer, characterized by the spread of malignant cells from its original site to other parts of the body, remains largely incurable, with cancer death rates declining by only 1.5 percent per year between years 2001 and 2017  [37, 42, 60].

This lack of progress in metastatic cancers is, in part, due to the standard of care in metastatic cancers, which typically applies a drug or drug combination at maximum tolerable dose (MTD), either continuously or in predefined treatment cycles [23, 25, 27, 28, 80]. The same regimen continues until there is unacceptable toxicity, unambiguous evidence of tumor progression, or cure. However, in metastatic cancers, cure is rare [21, 68, 70].

The goal of killing as many cancer cells as fast as possible may be evolutionarily unwise [22, 24, 28, 74, 75, 86]. This is because MTD imposes a very strong selection pressure for the evolution of treatment-induced resistance, which subsequently leads to treatment failure [1, 22, 24, 36, 42, 53, 61, 84, 85, 92].

Evolutionary cancer therapies (also known as adaptive therapies) provide an alternative to the standard of care [28, 31, 48, 56, 74, 75]. Evolutionary therapies aim to manage treatment-induced resistance in cancer cells by anticipating and steering their ecological and evolutionary dynamics. Such therapies integrate mathematical models, known cancer biology, and patient-specific data to improve care. Different therapy goals lead to different types of evolutionary therapy:

- Delaying progression or tumor burden stabilization: In situations where curative therapies are too risky or unavailable, evolutionary therapies strive to prolong the time to progression (clinical trials NCT02415621, NCT03511196, NCT05393791, NCT03543969, and NCT03630120) or, when possible, to stabilize the tumor burden by maintaining a tumor burden that is viable for the patient (clinical trial NCT05080556). In all named trials, the goal of treatment has shifted from “treat to eradicate” to a less ambitious but more attainable “treat to delay progression” or “treat to contain” [2, 17, 51, 84, 86]. The strategy of stabilization of an incurable disease has been motivated by the success of similar strategies outside of oncology, for instance, when treating human immunodeficiency virus (HIV) [13, 18, 49] and diabetes [14, 87].
- Cure: Recently, there have been attempts to cure metastatic cancers through extinction (or first strike—second strike) evolutionary therapy (clinical trials NCT04388839, and NCT04343365). This therapy aims at cure by applying a “first strike” treatment to decrease the tumor burden below a critical threshold, followed by “second strike” treatments aiming at cancer eradication [29, 32, 33].

The first and most well-known trial based on evolutionary therapy is Zhang et al.’s trial (NCT02415621), which aimed at delaying cancer progression. In this trial, patients with metastatic castrate-Resistant Prostate Cancer (mCRPC) were given abiraterone until their prostate specific antigen (PSA, a blood biomarker of tumor burden) dropped below 50% of its initial value [92, 93]. At this point, the abiraterone treatment was stopped and re-administered only when the PSA returned to its initial value. Then a new therapy cycle started. This led to cycles on and off abiraterone in response to the patients’ PSA levels. While Zhang et al.’s protocol eventually fails and progression occurs, it happens much later than with the standard of care [92, 93]. As of the time of writing, 12 of 16 patients have progressed (median time to progression of about 30 months compared to about 14 months for the standard of care), though after six years, four patients continue to cycle without any indication of disease progression [93]. Zhang et al.’s adaptive protocol is currently repeated in trial NCT05393791, with nearly 200 patients.

Despite the success of Zhang et al.’s trial, theory tells us that one could do even better for this target group of patients. Cunningham et al. [17] demonstrated with a variant of Zhang et al.’s model [92] that stabilization of the tumor burden may be possible in mCRPC. Further, they showed that even when it is unclear at what tumor burden one should stabilize, it is often possible to reach this stable tumor burden by gradually decreasing the dose whenever the tumor burden is decreasing, starting from the maximum tolerable dose [28]. Such a dose reduction therapy is currently being tested in patients with relapsed Platinum-sensitive High-Grade Serous or High-Grade Endometrioid Ovarian Cancer (clinical trial NCT05080556). In [17] and [77] a potentially even more effective approach is explored, a dose titration protocol, where the treatment starts with a very low dose, which is gradually increased whenever the tumor burden grows and decreased whenever it decays. These theoretical results and the mentioned ongoing clinical trial in ovarian cancer provide motivation for our present work, where we analyze what the best constant treatment dose is if cancer cannot be cured but can be stabilized at a viable tumor burden.

To do so, we use an evolutionary version of the leader-follower games originally introduced in economics to conceptualize interactions with an imbalance in power between firms in oligopolistic markets by the German economist von Stackelberg [38, 73]. Indeed, the physician and the cancer cells engage in a type of leader-follower game: The physician chooses a treatment dose to maximize an objective function expressing the patient’s quality of life and the cancer cells adapt to this treatment, in a way that affects the physician’s objective [75]. The difference with standard leader-follower games is that cancer cells are not modeled as rational players but as playing an evolutionary game that determines the cell types that emerge, their population size, and their evolutionary traits [5, 11, 20, 39, 81].

The combination of these two levels of interaction (between the physician and cancer cells and between the cancer cells themselves) results in a game that some of us have recently termed a Stackelberg evolutionary game [45, 66, 76]. Stackelberg evolutionary game theory applies to situations with a rational player (the physician in the case of cancer treatment) and evolutionary followers, which can be a community of populations, species, or types that evolve by natural selection (cancer cells in the case of cancer treatment). In these games, the rational player can act as the leader and anticipate and steer the eco-evolutionary dynamics of the followers, who adapt to the actions of the leader, according to the principles of natural selection.

Here, we extend the cancer model of Pressley et al. [61], who considered a polymorphic cancer cell population consisting of sensitive cells that do not evolve and resistant cells whose treatment-induced resistance is a quantitative evolving trait. Pressley et al. [61] assumed only density-dependent selection of cancer cells. Motivated by in vitro and in vivo studies that demonstrated the cancer cells’ frequency-dependent selection [26, 43, 57, 59, 79, 93], here we assume direct competition between the two cancer cell types, leading to frequency dependence. Moreover, we assume that the sensitive cancer cells have a larger competitive effect on the resistant cells than vice versa. Furthermore, as Pressley et al. [61], we assume a cost of resistance in the growth rate of resistant cells, i.e., the growth rate of resistant cancer cells decreases with the resistance level.

Pressley et al. [61] demonstrated that Zhang et al.’s adaptive therapy protocol prolonged the time to progression when compared to continuous therapy at maximum tolerable dose also in models with the treatment-induced resistance evolving as a quantitative trait. Here, we focus on a situation when cure is impossible, but the tumor burden can be stabilized at a viable level for the patient. For this case, we determine the optimal constant treatment dose maximizing the objective function of the physician, which is a function depending on the tumor burden, treatment toxicity, and the cancer cells’ level of treatment-induced resistance. This objective function captures the patient’s quality of life.

In what follows, we: (1) develop a model for the cancer’s eco-evolutionary dynamics, (2) expand it into a Stackelberg evolutionary game of cancer treatment, and (3) compare three different strategies that the physician might use to treat the patient (maximum tolerable dose, an ecologically enlightened strategy, and an evolutionarily enlightened strategy), while assuming the tumor burden has reached its equilibrium size. We conclude by highlighting the main outcomes and their relevance for the field of (mathematical) oncology, discussing model limitations, and proposing future research directions.

## Methods

Following [61], we consider a model with two cancer cell types: sensitive cells, whose resistance to treatment is minimal, and (potentially) resistant cells, which may adapt to evolutionary pressures by becoming more or less resistant to treatment. However, in contrast to Pressley et al. [61] we allow for differing magnitudes of inter- and intra-type competition between the two types of cancer cells. Furthermore, we determine for which treatment doses and treatment-induced resistance levels the tumor burden can be stabilized at levels viable for the patient. Stabilization in this context refers to employing a constant treatment dose that keeps the tumor burden at a progression-free/viable equilibrium size. When the tumor burden can be stabilized, we adopt a Stackelberg (leader-follower) evolutionary game theory approach [6, 66, 75, 76, 88]: The leader (physician) chooses a treatment dose with the aim of maximizing an objective function, which is a proxy for the patient’s quality of life and depends on the tumor burden, the resistance level of cancer cells, and the treatment dose.

The cancer cells adapt to treatment in two ways, which are easiest to conceptualize as a short-run and a long-run response (though, technically, our model does not assume a separation of time scales). In the short-run (ecological time scale), only the absolute and relative abundances of the two types of cancer cells evolve. In the longer-run (evolutionary time-scale), the resistance level of the resistant cells evolves as well. This resistance level may be seen as their (evolutionary) strategy. The function mapping a given treatment dose to the resistance level that evolves in response can be perceived as the cancer cells’ evolutionary response function, which is somewhat analogous to the best-response function of the follower in a standard Stackelberg game, in that it defines the response of the followers maximizing their objective with respect to their strategy. Drawing on this analogy, we call this function the cancer cells’ best-response function.

We consider three possible cases, leading to three potentially different therapeutic outcomes. In the first case, the physician just uses the maximum tolerable dose (MTD) of treatment. In the second case, the physician can observe the current resistance level of cancer cells. After a potential phase of “tatônnement”, where the physician adjusts the treatment dose based on the actual resistance level of cancer cells and the cancer cells evolve resistance in response to this updated treatment dose, this is expected to lead to a stable situation: the resistance level of resistant cells is a best response to the current treatment dose, and this treatment dose is a best response to the resistant cells’ current resistance level.

We refer to this stable situation as the (static) Nash equilibrium of the game. The corresponding strategy of the physician is called the Nash strategy.

In the third case, the physician also anticipates the evolutionary response of the cancer cells, meaning the physician knows in advance the best response of the cancer cells in terms of their resistance to all possible treatment doses that the physician can apply. This can lead to a stable outcome corresponding to a Stackelberg equilibrium of the leader-follower game, where the followers’ strategy is at an eco-evolutionary equilibrium, with respect to their strategy and population size. The corresponding strategy of the physician is their Stackelberg strategy, in accordance with the literature on Stackelberg games and our recent work [9, 45, 66, 69, 76]. The Stackelberg strategy fully exploits the leadership role of the physician and is, by construction, the leader’s best strategy [45, 75, 76].

When the tumor may be stabilized, we compare the outcomes corresponding to these three strategies: maximum tolerable dose, the ecologically enlightened/Nash strategy, and the evolutionarily enlightened/Stackelberg strategy.

### Model of Cancer Eco-Evolutionary Dynamics

We consider two distinct cancer cell populations: sensitive and resistant. We introduce frequency-dependent interactions between these two cell types by varying the intra- and inter-type competition coefficients. The treatment-induced resistance is modeled as a quantitative trait, meaning that this resistance exists on a continuum, and the resistant cells can exhibit some level of resistance $u_R(t)\ge 0$ that evolves over time *t* in response to therapy. While we assume that the sensitive cells do not evolve resistance ($u_S(t)=0$ for all *t*), we retain both $u_S$ and $u_R$ in our model, even though $u_S=0$ in the current scenario. This is done to allow for future extensions of this model where $u_S$ may reach different values. We model the eco-evolutionary dynamics of the cancer cells using a fitness-generating function, also known as a *G*-function [83]. The *G*-function defines how the fitness of a focal cancer cell using strategy *v* is influenced by the environment and the strategies and population sizes of the resident types. In particular, the *G*-function is crucial for determining the evolutionary dynamics for how the resistant strategy evolves over time. The resistance level evolves in the direction of the fitness gradient $\frac{\partial G}{\partial v},$ with respect to the focal individual’s strategy *v* [83]. This derivative is then evaluated at the current resident strategy $u_R,$ leading to an equation defining the evolutionary dynamics for the resident strategy itself [61, 83]. The rate at which the resistance strategy changes is scaled by an evolutionary speed term $\sigma ,$ which we assume constant for simplicity. The exact value of $\sigma$ does not influence the equilibrium outcomes presented in this paper.

The eco-evolutionary dynamics of the two populations of cancer cells are as follows:

1 $$\begin{aligned} \frac{\textrm{d} x_R}{\textrm{d} t}&=\,x_R\,G\left( v,\textbf{u},\textbf{x},m\right) \bigg |_{v=u_R},\end{aligned}$$

2 $$\begin{aligned} \frac{\textrm{d} u_R}{\textrm{d} t}&=\,\sigma \, \frac{\partial G \left( v,\textbf{u},\textbf{x},m\right) }{\partial v}\bigg |_{v=u_R},\end{aligned}$$

3 $$\begin{aligned} \frac{\textrm{d} x_S}{\textrm{d} t}&=x_S \left( r(u_S)\left( 1-\frac{\alpha _{SS}\, x_S+\alpha _{SR}\, x_R}{K}\right) -d-\frac{m}{k+bu_S} \right) ,\end{aligned}$$

4 $$\begin{aligned} u_S&=0, \end{aligned}$$

where we assume that the *G*-function has the following form:

5 $$\begin{aligned} G(v, \textbf{u}, {\textbf {x}},m)=r(v)\left( 1-\frac{\alpha _{RS}\, x_S+\alpha _{RR}\, x_R}{K}\right) -d-\frac{m}{k+bv}, \end{aligned}$$

and that the vectors $\textbf{u} = (u_R, u_S)^\top$ and $\textbf{x} = (x_R, x_S)^\top$ capture the resistance strategies and population sizes of the two cancer cell populations, respectively. Treatment-induced resistance may come at a cost [74], which may make resistant cells less fit in the absence of treatment. In (5), we assume a cost of resistance in the intrinsic growth rate. More specifically, $r(v)=r_{\max }{e}^{-g\,v}$, where *g* determines the magnitude of the effect of resistance on the growth rate. The intra-type competition effects are given by $\alpha _{SS}$ and $\alpha _{RR}$, while $\alpha _{RS}$ and $\alpha _{SR}$ are the inter-type competition coefficients. Parameter *K* denotes the carrying capacity of cancer cells, while parameter *d* is the natural death rate of cancer cells. In our model, we assume that the physician applies a treatment dose $m(t)\in [0,1]$ at time $t\ge 0$, where $m(t)=0$ corresponds to no dose and $m(t)=1$ to MTD. We assume that drug efficacy increases with *m* and decreases with the focal cell’s resistance strategy *v*, its innate resistance *k*, and the benefit *b* of the resistance level.

Parameter values, except the cost of resistance’s magnitude *g* and inter-type competition coefficients $\alpha _{RS}$ and $\alpha _{SR}$, are taken from [61]. The ratio of the inter-type competition coefficients is taken from [93]. Parameters and their values are summarized in Table 1.

**Table 1:** Variables and parameter values of the game [61, 93]

Variables	Meaning	Feasible values
*x*	Cancer cell population	[0, *K*]
$x_S$	Sensitive population	[0, *K*]
$x_R$	Resistant population	[0, *K*]
$u_S$	Resistance strategy of sensitive cells	0
$u_R$	Resistance strategy of resistant cells	$\mathbb {R}_+$
*v*	Resistance strategy (focal individual)	$\mathbb {R}_+$
*m*	Treatment dose	[0, 1]

Parameters		Values
$r_{\max }$	Intrinsic growth rate of the cancer cells	0.45
*d*	Intrinsic death rate	0.01
*K*	Carrying capacity	10000
*g*	Magnitude of cost of resistance	0.8
*k*	Innate cell immunity	2
*b*	Magnitude of resistance benefit	10
$\sigma$	Evolutionary speed of resistant cells	1
$\delta$	Progression threshold (fraction of *K*)	0.7
$\alpha _{RS}$	Inter-type competition coefficient	0.9
$\alpha _{SR}$	Inter-type competition coefficient	0.15
$\alpha _{SS}$	Intra-type competition coefficient	1
$\alpha _{RR}$	Intra-type competition coefficient	1

### Viability Analysis of the Eco-Evolutionary Equilibria of Cancer Dynamics

The time to progression corresponds to the moment when the tumor burden exceeds a predefined fraction  $\delta \in (0,1]$ of the carrying capacity. We set this $\delta$ to 0.7 as in [61]. Our first objective is to identify treatment doses and treatment-induced resistance levels for which it is possible to maintain the tumor burden below the progression threshold $\delta K$.

**Fig. 1:**
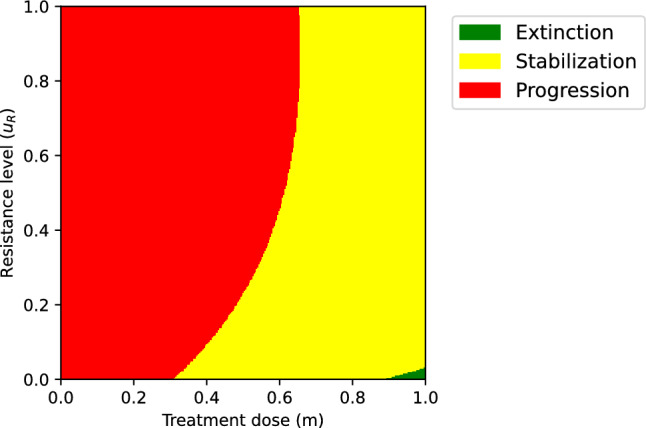
The equilibrium population size. We identify three possible regions: The green area $\mathcal {G}$ corresponds to combinations of *m* and $u_R$ for which $x^*(m,u_R) = 0$, i.e., the region where cancer is cured; the red area $\mathcal {R}_{\delta }$ corresponds to combinations of *m* and $u_R$ for which $x^*(m,u_R)> \delta K$, i.e., the region where the tumor burden is too high, and the disease progresses; the yellow area $\mathcal {Y}_{\delta }$ corresponds to combinations of *m* and $u_R$ for which the tumor burden stabilizes at a nonzero but viable level, and the corresponding values of the objective function will matter. Parametrization: $\delta =0.7$, $r_{\max }=0.45$, $g=0.8$, $K=10000$, $d=0.01$, $k=2$, $b=10$, $\alpha _{SS}=\alpha _{RR}=1$, $\alpha _{SR}=0.15$, $\alpha _{RS}=0.9$ (Color figure online)

The sizes of sensitive and resistant cancer cell populations at the ecological equilibria $x_R^*(m,u_R)$ and $x_S^*(m,u_R)$ at any given resistance level are found by solving $\frac{\textrm{d} x_R}{\textrm{d} t}=0$ and $\frac{\textrm{d} x_S}{\textrm{d} t}=0$, respectively. The tumor burden $x^*(m,u_R)$ is defined as $x^*(m,u_R)=x_R^*(m,u_R)+ x_S^*(m,u_R).$ The extinction, stabilization, and progression regions are defined as follows:

- *Extinction region*: all $(m,u_R)$-pairs for which the cancer population eventually goes extinct, i.e., where the tumor burden at equilibrium $x^*(m,u_R)$ is zero (shown in green in Fig. 1):
  $$\mathcal {G}=\{(m,u_R)\in [0,1]\times \mathbb {R}_+: x^*(m,u_R) = 0\}.$$
- *Stabilization region*: all $(m,u_R)$-pairs for which the tumor burden at equilibrium $x^*(m,u_R)$ is positive, but does not exceed $\delta K$ (shown in yellow in Fig. 1):
  $$\mathcal {Y}_{\delta }=\{(m,u_R)\in [0,1]\times \mathbb {R}_+: 0<x^*(m,u_R) \le \delta K\}.$$
- *Progression region*: all $(m,u_R)$-pairs for which the tumor burden progresses, i.e., for which the tumor burden at equilibrium $x^*(m,u_R)$ is higher than the progression threshold $\delta K$ (shown in red in Fig. 1):
  $$\mathcal {R}_{\delta }=\{(m,u_R)\in [0,1]\times \mathbb {R}_+: x^*(m,u_R)>\delta K\}.$$

Fig. 1 shows different values of the ecological equilibrium $x^*(m,u_R)$ for different *m* and $u_R$ values, determining the three regions $\mathcal {G},$ $Y_{\delta },$ and $R_{\delta }$ for the particular parametrization of the model.

According to (2), the treatment-induced resistance ${u_R}$ under a particular treatment dose *m* eventually evolves towards an evolutionarily stable strategy (ESS) $u_R^*=u_R^*(m)$. At eco-evolutionary equilibrium, the cancer population is at its ecological equilibrium and $u_R=u_R^*.$ By definition, when the population is at the eco-evolutionary equilibrium, the ESS strategy maximizes *G*  [3, 83] and the first order condition applies:

6 $$\begin{aligned} \frac{\partial G(v, \textbf{u}^*, {\textbf {x}}^*,m)}{\partial v} \bigg |_{v=u^*_R}=0{,} \end{aligned}$$

whenever this derivative exists.

We assume that it is possible to cure or contain the disease with a constant treatment dose if values of *m* exist for which the pair $(m,u_R^*(m))$ lies in the extinction or stabilization region, respectively. Otherwise, progression under a constant treatment dose is inevitable.

### Expanding the Model into a Stackelberg Evolutionary Game

In the previous section, we introduced an evolutionary game between different types of cancer cells. Here, we extend this game into a Stackelberg evolutionary game, where the physician as the leader maximizes the patient’s quality of life through selecting a particular treatment dose. This quality of life is captured in an objective function, defined for treatment doses and resistance levels where cure is unachievable but stabilization is possible. By maximizing such an objective function with respect to the treatment dose, we determine which of the treatment strategies leading to a viable tumor burden is the most desirable for the patient. Our objective function depends on the cancer cell population, the toxicity due to the treatment dose, and the treatment-induced resistance of the cancer cells. We assume that the objective function decreases when the tumor burden increases, as the patient might experience pain or other side effects  [41, 52]. Similarly, the objective function decreases with the treatment dose, because of the increased treatment-induced toxicity. Moreover, we assume that the objective function declines with cancer’s resistance level, as more resistant cells might give rise to secondary tumors and side effects [54, 82], which decrease the patient’s quality of life. Besides, the resistance level could be associated with the Warburg effect, which may alter the micro-environment to be more suitable for cancer cell proliferation [47]. Here, we introduce the following objective function *Q*:

7 $$\begin{aligned} Q(m,u_R,x^*(m,u_R))= {\left\{ \begin{array}{ll} Q^{\max }-c_1\big (\frac{x^*(m,u_R)}{K}\big )^2-c_2u_R^2-c_3m^2,& (m,u_R)\in \mathcal {Y}_{\delta },\\ \text {undefined,} & \text {elsewhere}. \end{array}\right. } \end{aligned}$$

In (7), $Q^{\max }$ represents the maximum value of the objective function, while the weights $c_1$, $c_2$, and $c_3$ (where $c_1+c_2+c_3=1$, $c_1, c_2, c_3\in [0,1]$) determine the impact of tumor burden, treatment-induced resistance level, and treatment toxicity on the patient’s quality of life, respectively.

**Fig. 2:**
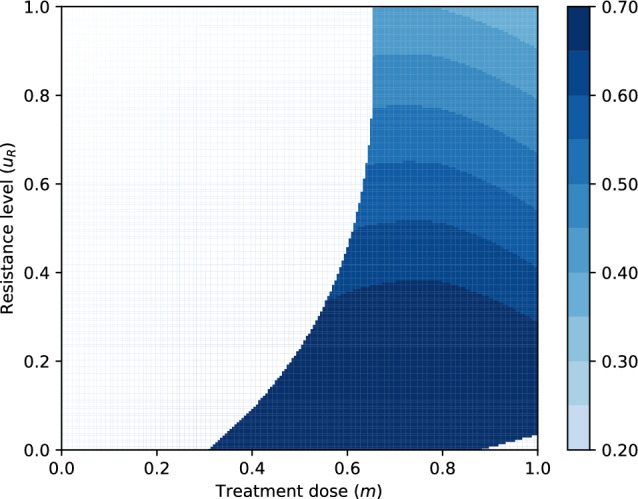
The physician’s objective function as a function of the tumor burden, the treatment dose and the resistance level (7). The white space illustrates the region where the objective function is undefined. Parametrization: $\delta =0.7$, $r_{\max }=0.45$, $g=0.8$, $K=10000$, $d=0.01$, $k=2$, $b=10$, $\alpha _{SS}=\alpha _{RR}=1$, $\alpha _{SR}=0.15$, $\alpha _{RS}=0.9$, $\sigma =1$, $Q^{\max }=1$, $c_1=0.5,$ $c_2=0.25,$ $c_3=0.25$

Figure 2 depicts the objective function (7) for a particular parametrization.

In those cases where the tumor burden can be stabilized, but cure is impossible, we compare the following treatment strategies and their effects on the physician’s objective function (see also Table 2):

- Ecologically enlightened strategy (or Nash strategy): The physician considers the ecological but not the evolutionary effects of treatment. For a fixed resistance level $u_R$, the best response of the physician would be the treatment dose:
  8 $$\begin{aligned} m^*(u_R)=\arg \mathop {\max }\limits _{m} Q(m,u_R,x^*(m,u_R)). \end{aligned}$$
  Besides, for a given treatment dose *m*, the cancer cells’ resistance evolves to their ESS given by (6). We refer to the stable situation this may lead to as the Nash equilibrium of the game (meaning the Nash equilibrium of the underlying simultaneous-move game). It lies at the intersection of the cancer cells’ evolutionary response (ESS strategy) curve $u_R^*(m)$ and the physician’s best response curve $m^*(u_R)$. This Nash equilibrium is only meaningful if these curves intersect within the stabilization region. When this is the case, we denote by $m_N$ the physician’s Nash strategy and by $u_R^*(m_N)$ the cancer cells’ Nash strategy.
- Evolutionarily enlightened strategy (or Stackelberg strategy): The physician anticipates the ecological and evolutionary response of the cancer cells to therapy. It is defined as
  9 $$\begin{aligned} m_{S}=\arg \max _{m} Q(m,u_R^*(m),x^*(m,u_R^*(m))). \end{aligned}$$
  The cancer cells’ strategy at the Stackelberg equilibrium is given by their ESS $u_R^*(m_S)$.

For some parametrizations of our model, the MTD, i.e., $m=1,$ can also stabilize the tumor burden. In such a case, the physician’s objective function (7) is defined for $m=1$ as well. If that is the case, we can compare the outcomes of ecologically and evolutionarily enlightened treatment strategies with the physician’s objective function under MTD.

**Table 2:** Four notions of equilibria utilized in this paper

Ecological equilibrium	The populations of sensitive and resistant cancer cells are not changing ($\dot{x}_S=0$ and $\dot{x}_R=0$) given the current resistance level $u_R$ and the current treatment dose *m*.
Eco-evolutionary equilibrium	The cancer cells are at the ecological equilibrium, and the resistance level of the resistant cells is not changing ($\dot{u}_R=0$).
(static) Nash equilibrium	The cancer cells are at an eco-evolutionary equilibrium while the treatment dose is at the best response to the current resistance level.
Stackelberg equilibrium	Cancer cells are at an eco-evolutionary equilibrium, and the physician maximizes the objective function knowing the evolutionary response of cancer cells.

## Results

We first calculate the ecological equilibria of cancer cells. We then calculate the physician’s Nash and Stackelberg strategies and the game’s corresponding outcomes in terms of the objective function of the physician (7). When the equilibrium population size lies in the patient’s stabilization region, we compare the MTD, Stackelberg and Nash outcomes of the game. In Appendix A, we illustrate the basins of attraction of these equilibria. Competition coefficients $\alpha _{SS}$ and $\alpha _{RR}$ are set to 1 as in [61], which is a common assumption in the ecology literature [12, 26, 40, 83].

### Ecological Equilibria of Cancer Cells

The ecological equilibria of the cancer cells can be found by setting $\frac{\textrm{d} x_R}{\textrm{d} t}$ and $\frac{\textrm{d} x_S}{\textrm{d} t}$ to zero. With

$$\begin{aligned} \hat{x}_S(m,u_R)&{\mathop {=}\limits ^\textrm{def}} \frac{K}{r_{\max }(1-\alpha _{SR}\alpha _{RS})}\left( \frac{\alpha _{SR}\,m\,\textrm{e}^{g\,u_R}}{k+b\,u_R}-\frac{m}{k}+\alpha _{SR}\,d\,\textrm{e}^{g\,u_R}\right) \\\nonumber&\quad +\frac{K\left( r_{\max }-d-\alpha _{SR}\,r_{\max }\right) }{r_{\max }(1-\alpha _{SR}\alpha _{RS})},\\ \hat{x}_R(m,u_R)&{\mathop {=}\limits ^\textrm{def}} \frac{K}{r_{\max }(1-\alpha _{SR}\alpha _{RS})}\left( -\frac{m\,\textrm{e}^{g\,u_R}}{k+b\,u_R}+\frac{\alpha _{RS}\,m}{k}-d\,\textrm{e}^{g\,u_R}\right) \\\nonumber&\quad +\frac{K\left( r_{\max }-\alpha _{RS}r_{\max }+\alpha _{RS}\,d\right) }{r_{\max }(1-\alpha _{SR}\alpha _{RS})},\end{aligned}$$

we obtain the following ecological equilibria $(x_S^*(m, u_R),x_S^*(m, u_R))$:

10 $$\begin{aligned} (x_S^*(m, u_R),x_R^*(m, u_R))&{\mathop {=}\limits ^\textrm{def}} \left\{ \begin{array}{l} (0,0) , \quad \quad \quad \quad \,\,\,\quad \text{ if } \quad \quad \hat{x}_S\le 0,\, \hat{x}_R \le 0, \\ \left( \max \{\bar{x}_S,0\},0\right) , \quad \text{ if } \,\,\,\,\,\quad \hat{x}_S \ge 0,\,\hat{x}_R \le 0, \\ \left( 0,\max \{\bar{x}_R,0\}\right) , \quad \text{ if } \,\,\,\,\,\quad \hat{x}_S \le 0,\, \hat{x}_R \ge 0, \\ \left( \hat{x}_S,\hat{x}_R\right) ,\quad \quad \quad \quad \,\,\text{ if } \quad \,\,\,\,\hat{x}_S> 0,\, \hat{x}_R > 0, \end{array} \right. \end{aligned}$$

with

$$\begin{aligned} \bar{x}_S&=K\left( 1-\frac{d+\frac{m}{k}}{r_{\max }}\right) ,\\ \bar{x}_R&=K\left( 1-\frac{d+\frac{m}{k+b\,u_R}}{r_{\max }} \textrm{e}^{g\,u_R}\right) . \end{aligned}$$

**Fig. 3:**
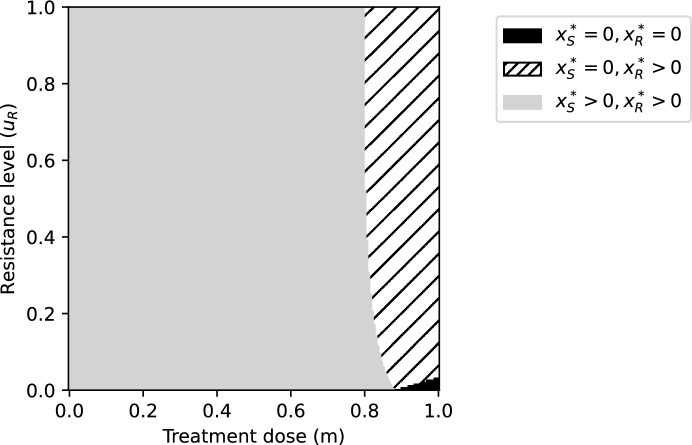
The $x^*_S(m,u_R)$ and $x^*_R(m,u_R)$ values corresponding to different *m* and $u_R$ values. We identify three possible regions: The black area corresponds to $(m,u_R)\in [0,1]\times [0,1]$ where $x^*_S(m,u_R)=x^*_R(m,u_R)=0$, the dashed area corresponds to $(m,u_R)\in [0,1]\times [0,1]$ where $x^*_S(m,u_R)=0,$ $x^*_R(m,u_R)>0$, and the gray area indicates the region $(m,u_R)\in [0,1]\times [0,1]$ where both populations coexist, i.e., $x^*_S(m,u_R)>0,$ $x^*_R(m,u_R)>0$. Parametrization: $\delta =0.7$, $r_{\max }=0.45$, $g=0.8$, $K=10000$, $d=0.01$, $k=2$, $b=10$, $\alpha _{SS}=\alpha _{RR}=1$, $\alpha _{SR}=0.15$, $\alpha _{RS}=0.9$

In Fig. 3, we illustrate the areas in the $(m,u_R)$-plane with different types of ecological equilibria (10).

Extending the Pressley et al. [61] model to include competition coefficients creates a non-monotonic relation between the total population size at the ecological equilibrium and treatment dose. In Pressley et al. [61] model, the total equilibrium population size decreases as the treatment dose increases, which means that lower constant treatment doses will also fail if MTD fails. Therefore, we consider the extended model more realistic for many cancers and treatments. The cell population is considered extinct in areas where $x_S^*= 0$ and $x_R^*= 0$.

### The Best Response Curves of Cancer and the Physician

The best response curve of the resistant cancer cell population (ESS curve) is determined using (6). With $\displaystyle \hat{u}_R(m){\mathop {=}\limits ^\textrm{def}}-\frac{k}{b}-\frac{m}{2bd}+\sqrt{\frac{m^2}{4b^2d^2}+\frac{m}{bdg}},$

11 $$\begin{aligned} u_R^*(m)=\left\{ \begin{array}{l}0, \quad \quad \quad \,\,\text{ if } \quad \hat{u}_R(m) < 0, \\ \hat{u}_R(m), \quad \text{ otherwise. } \end{array} \right. \end{aligned}$$

Note that $\hat{u}_R(m)$ increases with *m*. The best response curve $m^*(u_R)$ of the leader is determined by maximizing their objective function $Q(m,u_R^*(m),x^*(m,u^*_R(m)))$ with respect to *m*. Note that the objective function is differentiable and concave in *m*, and, therefore, $m^*(u_R)$ is calculated by setting the first derivative of the objective function to zero and projection on [0, 1] whenever the result falls outside of this interval. Letting ${A = \frac{(1-\alpha _{SR})\textrm{e}^{gu_R}}{k+bu_R}+\frac{1-\alpha _{RS}}{k}}$ and

$${\hat{m}(u_R){\mathop {=}\limits ^\textrm{def}}\small {\frac{\textstyle c_1 A \left( r_{\max }(2-\alpha _{RS}-\alpha _{SR})-(1-\alpha _{RS})d-(1-\alpha _{SR})d\textrm{e}^{gu_R}\right) }{\displaystyle c_1\, A^2 + c_3\,r_{\max }^2(1-\alpha _{SR}\alpha _{RS})^2}}, }$$

the leader’s best response then reads as

12 $$\begin{aligned} m^*(u_R) = \left\{ \begin{array}{l}0, \quad \quad \quad \,\,\text{ if }\quad \hat{m}(u_R)<0,\\ \hat{m}(u_R),\quad \text{ if } \quad \hat{m}(u_R)\in [0,1],\\ 1,\quad \quad \quad \,\,\,\text{ otherwise. } \end{array} \right. \end{aligned}$$

The parameter $c_2$ (determining the impact of treatment-induced resistance on the objective function) has no effect on the leader’s best response curve and, therefore, does not influence the Nash solution. However, the parameter $c_2$ affects the Stackelberg solution.

### Identifying the Nash and Stackelberg Equilibria

The Nash equilibrium lies at the intersection of the best response curves of cancer cells and the physician, defined by (11) and (12), respectively. The Stackelberg equilibrium is calculated numerically through  (9). It corresponds to the point on the cancer’s best response curve that maximizes the physician’s objective function.

**Fig. 4:**
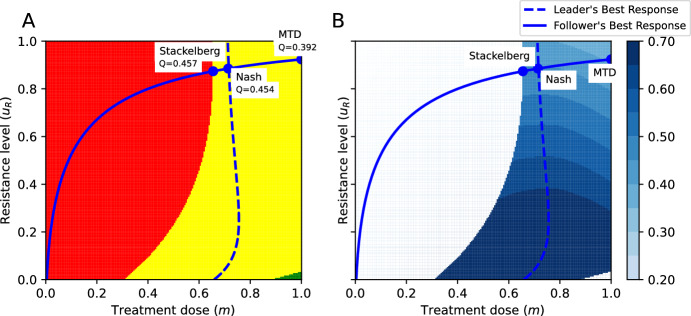
The outcomes of the MTD, ecologically enlightened (Nash), and evolutionarily enlightened (Stackelberg) strategies of the physician in the SEG against cancer: The yellow and red regions represent combinations of $u_R$ and *m* leading to tumor stabilization and progression, respectively. **A** Illustration of the outcomes of the Stackelberg, Nash, and MTD treatment strategies and corresponding values of the objective function. **B** The level curves of the objective function and outcomes of the Nash, Stackelberg, and MTD treatment strategies. Parametrization: $\delta =0.7$, $r_{\max }=0.45$, $g=0.8$, $K=10000$, $d=0.01$, $k=2$, $b=10$, $\alpha _{SS}=\alpha _{RR}=1$, $\alpha _{SR}=0.15$, $\alpha _{RS}=0.9$, $Q^{\max }=1$; $c_1=0.5,$ $c_2=0.25,$ $c_3=0.25$ (Color figure online)

Figure 4A shows a particular parametrization of the model for which the MTD and the Nash and Stackelberg equilibria all lie in the stabilization region. The physician’s best response, calculated through (12), is shown as a dashed line. Figure 4B shows that for this parametrization, the evolutionarily enlightened (Stackelberg) strategy leads to the highest value of the objective function, followed by the ecologically enlightened (Nash) strategy, while MTD leads to the lowest value of the objective function. Moreover, the evolutionarily enlightened (Stackelberg) strategy corresponds to both a lower treatment dose/toxicity and lower treatment-induced resistance than the ecologically enlightened (Nash) strategy and MTD.

**Fig. 5:**
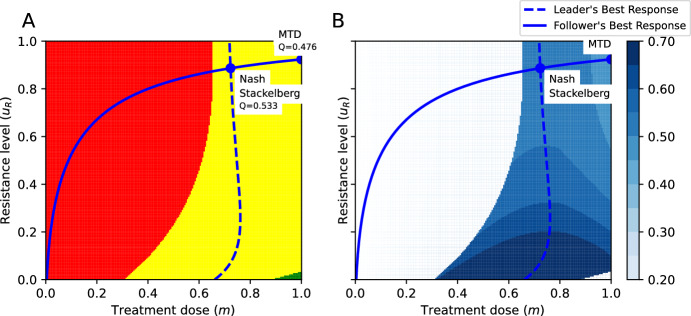
The outcomes of the MTD, ecologically enlightened (Nash) and evolutionarily enlightened (Stackelberg) strategies of the physician in the SEG against cancer: The yellow and red regions represent tumor burden stabilization at a safe level and progression, respectively. **A** The Nash and Stackelberg strategies coincide. **B** The values of the physician’s objective function coincide with the Nash and Stackelberg strategies and are better than that of MTD. Parametrization: $\delta =0.7$, $r_{\max }=0.45$, $g=0.8$, $K=10000$, $d=0.01$, $k=2$, $b=10$, $\alpha _{SS}=\alpha _{RR}=1$, $\alpha _{SR}=0.15$, $\alpha _{RS}=0.9$, $Q^{\max }=1$; $c_1=0.67,$ $c_2=0,$ $c_3=0.33$ (Color figure online)

The Nash and Stackelberg equilibria of a Stackelberg evolutionary game can coincide under certain conditions, for instance, if the leader’s strategy does not affect the evolution of the resistance level of the cancer cells, as proven by  [76].^1^ Fig. 5 illustrates the case where the Nash and Stackelberg solutions coincide, due to the fact that the objective function does not include treatment-induced resistance [76]. However, even in this situation, the MTD results in a lower value of the objective function than the Nash and Stackelberg strategies. In appendix A, the local stability of the eco-evolutionary equilibria is determined through numerical analysis of the Jacobian matrix. Furthermore, in this appendix, we illustrate the domain of attraction of the Nash equilibrium.

## Discussion

Cancer treatment is a Stackelberg (or leader-follower) evolutionary game. Recent works, including  [45, 75, 76, 88], suggested that physicians should exploit the advantages of their leadership role in this game. This is because the physician, unlike the cancer cells, can anticipate and steer the cancer’s eco-evolutionary response to treatment, while the cancer cells can only adapt to the current and past physician’s actions. Staňková et al. [75] proposed that in order to utilize their leadership role fully, the physician needs to (i) set the treatment goal, as different treatment goals will correspond to different treatment strategies, (ii) introduce a resistance management plan, and (iii) perform after-action reports, adjusting assumptions and parameters based on how different patients respond to the treatment (see also [93]).

In this work, we focused on a specific treatment goal, finding the constant treatment dose maximizing the physician’s objective function once the tumor burden can be stabilized at a level viable for the patient. We did so by utilizing the Stackelberg evolutionary game framework where cancer is modeled as an evolutionary game, extending the polymorphic cancer model by Pressley et al. [61]. In their paper, Pressley et al. compared the time to progression of Zhang et al. [92]’s protocol to that of the maximum tolerable dose. They demonstrated that, while the adaptive protocol always extended the time to progression, in some cases, this improvement was rather small.

Rather than analyzing the transient phase of controlling the tumor burden and prolonging the time to progression [16, 17, 35, 61], here we have focused on finding a constant treatment dose maximizing the physician’s objective function when the tumor burden can be stabilized [8]. This physician’s objective function depends on the tumor burden, the treatment toxicity, and the treatment-induced resistance in cancer cells. The weights in this objective function can be adjusted to capture the importance of the tumor burden, resistance level, and treatment toxicity for each patient, in line with patient-centered care [65]. Subsequently, we analyzed the impact of different treatment strategies in terms of this objective function: MTD, an ecologically enlightened therapy, and an evolutionarily enlightened therapy.

We have shown that the evolutionarily enlightened therapy leads to at least as high values of the objective function as the ecologically enlightened therapy, while the MTD leads to the lowest objective function values. For most parametrizations, the ecologically enlightened therapy leads to a higher treatment dose than the evolutionarily enlightened therapy, while both are less toxic than MTD.

For some parametrizations, the evolutionarily enlightened treatment corresponding to the Stackelberg strategy of the Stackelberg evolutionary game of cancer treatment leads to an outcome that is at the boundary of the progression region (Fig. 4). This means that a small deviation in estimating the cancer cells’ response would lead to growth of the tumor burden. However, upon observation of this cancer growth, the treatment dose could be increased a little, and the cancer would be stabilized again; thus, aiming at the Stackelberg strategy is still the best option.

In general, reaching any equilibrium requires frequent measurements of the tumor volume and its composition and depends on many factors, such as the speed of the cancer’s response to treatment. However, the physician may still be able to find the Stackelberg equilibrium by dose de-escalation, meaning starting from the MTD and applying small adjustments to the dose until the desired equilibrium is reached, as suggested by, among others, Enriquez-Navas et al. [23] and Cunningham et al. [17]. Another effective strategy may be starting from a minimal effective dose and gradually increasing it until an equilibrium is reached, which seems even more effective in a model similar to that of Zhang et al. [92]. This strategy has not yet been tested in clinical trials but may have potential [17, 78].

The model that we studied included treatment-induced resistance as a quantitative trait. Another option is to model resistance as a qualitative trait [4, 7, 10, 11, 35, 43, 55, 67, 71, 91]. Our model can be also extended to include several quantitative traits, evolving in response to multiple drugs and therapeutics [45, 64, 76]. Of interest in such models are cross-sensitivities [89, 90], co-resistance [58], and more possible effective treatment strategies available in a multi-drug setting, such as evolutionary double bind therapy [15, 30, 50].

In our model, the cost of resistance is associated with the growth rate of the resistant cancer cell type. Alternatively, one could consider models where either the carrying capacity [61] or the competition coefficients [83] explicitly depend on the resistance level.

In accordance with some parametrizations of the model presented here, there are cancers for which MTD can effectively stabilize tumors [6, 34, 44]. Also, there are metastatic cancers where we may be able to aim for a more ambitious treatment goal than tumor stabilization, namely for cure [22, 32]. For example, in multiple myeloma, there is a discussion on when to aim for a cure instead of containment. Strategies for these two goals differ substantially [19, 62, 63] and game-theoretic models fitted with patient data may help us to find a suitable treatment goal, corresponding evolutionary therapy, and predict the patient/tumor response to such therapy.

Our future research, therefore, will focus on the analysis of the properties of a wider class of game-theoretical models based on classical dynamics [34, 46], extended into a game-theoretic setting, and validating these models through in-vitro and in-vivo data, similarly to how it was done for other cancer models [44, 71, 72]. The ultimate goal is to analyze multiple dynamics that fit the data well, evaluate alternative hypotheses and models, and propose suitable treatment goals and evolutionary therapies to improve patient quality of life and survival.

## Figures and Tables

**Fig. 6:**
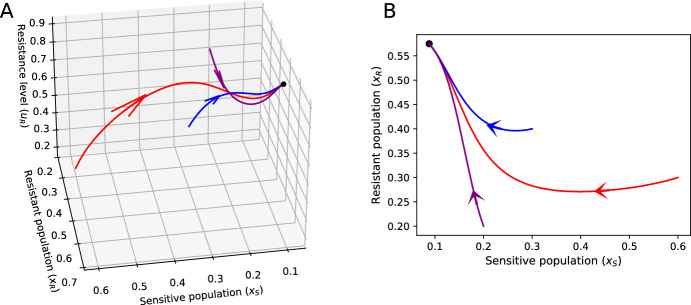
Eco-evolutionary dynamics of cancer while applying the Nash strategy dose ($m_N=0.723$), starting from 3 different initial conditions, with $(x_S(0), x_R(0), u_R(0))$ equal to (0.5, 0.1, 0.2),  (0.3, 0.4, 0.5),  and (0.2, 0.2, 0.7),  respectively. The eco-evolutionary dynamics starting from these three initial conditions are denoted in red, blue, and purple, respectively. We see how the eco-evolutionary dynamics starting at these initial conditions evolve in time and converge to the Nash equilibrium. **A** The eco-evolutionary dynamics. **B** The ecological dynamics. Parametrization: $\delta =0.7$, $r_{\max }=0.45$, $g=0.8$, $K=10000$, $d=0.01$, $k=2$, $b=10$, $\alpha _{SS}=\alpha _{RR}=1$, $\alpha _{SR}=0.15$, $\alpha _{RS}=0.9$, $\sigma =1$ (Color figure online)

## Data Availability

Not applicable.

## Appendix A: Basins of Attraction of Eco-Evolutionary Equilibria

The determination of the local stability of the eco-evolutionary equilibria involves the analysis of the Jacobian matrix at these equilibria. We confirm the local stability of the equilibria for treatment dosage *m* within 0.6 to 0.8 numerically, through analysis of the eigenvalues of the corresponding Jacobian matrix. We employ numerical modeling to demonstrate the domain of attraction for the Stackelberg and Nash equilibria (Fig. 5). Similarly, we can investigate the basins of attraction for other derived equilibria, such as those in Fig. 4. The sensitive and resistant populations ($x_S$ and $x_R$) are normalized (assuming $K=1$), to make the visualizations more informative. We consider initial values $x_S(0)$, $x_R(0)$, $u_R(0)$ as points in the grid $\varvec{\ell }\times \varvec{\ell }\times \varvec{\ell }$ where $\varvec{\ell }=\{0.1, 0.2, 0.3, \ldots , 1\},$ and $x_S(0)+x_R(0)<1$. The model converges to the eco-evolutionary equilibrium for all initial conditions within this grid. As an example, we illustrate this convergence to the equilibria from 3 different initial values. In Fig. 6, we illustrate how cancer eco-evolutionary dynamics starting in the stabilization region remain in the same region when reaching the Nash equilibrium. As we have not proven this for all possible parametrizations, there may be parametrizations with which the evolutionary dynamics leave the stabilization region before reaching equilibrium.

## Footnotes

1 The analogy with a standard leader-follower game is that if the follower has a strictly dominant action, then the outcomes of the subgame perfect equilibrium of the leader-follower game and of the Nash equilibrium of the simultaneous move game coincide.

## Code availability

https://github.com/HastiGarjani/Stackelberg-Evolutionary-Game-in-Cancer.git

## References

[1] Aguadé-Gorgorió G, Anderson AR, Solé R (2024) Modeling tumors as complex ecosystems. iScience 27(9):110699. 10.1016/j.isci.2024.110699 39280631 10.1016/j.isci.2024.110699 PMC11402243

[2] Alvarez FE, Viossat Y (2024) Tumor containment: a more general mathematical analysis. J Math Biol 88(4):41 38446165 10.1007/s00285-024-02062-3

[3] Apaloo J, Brown J, Vincent T (2009) Evolutionary game theory: ESS, convergence stability, and NIS. Evol Ecol Res 11(4):489–515

[4] Archetti M (2016) Cooperation among cancer cells as public goods games on Voronoi networks. J Theor Biol 396:191–203. 10.1016/j.jtbi.2016.02.027 26930167 10.1016/j.jtbi.2016.02.027

[5] Archetti M, Pienta KJ (2019) Cooperation among cancer cells: applying game theory to cancer. Nat Rev Cancer 19(2):110–117 30470829 10.1038/s41568-018-0083-7 PMC8557269

[6] Ardévol Martinez V, Salvioli M, Ghaffari Laleh N et al (2023) Improving mathematical models of cancer by including resistance to therapy: a study in non-small cell lung cancer. bioRxiv. 10.1101/2021.10.29.466444

[7] Basanta D, Simon M, Hatzikirou H et al (2008) Evolutionary game theory elucidates the role of glycolysis in glioma progression and invasion: game theory and the role of glycolysis. Cell Prolif 41(6):980–987. 10.1111/j.1365-2184.2008.00563.x 19040573 10.1111/j.1365-2184.2008.00563.x PMC6495695

[8] Basar OY, Mohammed S, Qoronfleh MW et al (2024) Optimizing cancer therapy: a review of the multifaceted effects of metronomic chemotherapy. Front Cell Dev Biol 12:1369597 38813084 10.3389/fcell.2024.1369597 PMC11133583

[9] Başar T, Olsder GJ (1998) Dynamic noncooperative game theory. SIAM

[10] Bayer P, Brown JS, Staňková K (2018) A two-phenotype model of immune evasion by cancer cells. J Theor Biol 455:191–204. 10.1016/j.jtbi.2018.07.014 30031001 10.1016/j.jtbi.2018.07.014

[11] Bayer P, Gatenby RA, McDonald PH et al (2022) Coordination games in cancer. PLOS ONE 17(1):e0261578. 10.1371/journal.pone.0261578 35061724 10.1371/journal.pone.0261578 PMC8782377

[12] Berryman AA (1992) The origins and evolution of predator-prey theory. Ecology 73(5):1530–1535. 10.2307/1940005 (https://esajournals.onlinelibrary.wiley.com/doi/10.2307/1940005)

[13] Blumenthal GM, Birnkrant D, Pazdur R (2018) Leveraging the success of HIV drug development paradigms for cancer. Clin Cancer Res 24(11):2491–2492. 10.1158/1078-0432.CCR-18-0544 10.1158/1078-0432.CCR-18-0544 29490988

[14] Chun J, Strong J, Urquhart S (2019) Insulin initiation and titration in patients with type 2 diabetes. Diabetes Spectr 32(2):104–111. 10.2337/ds18-0005 31168280 10.2337/ds18-0005 PMC6528396

[15] Cunningham JJ, Gatenby RA, Brown JS (2011) Evolutionary dynamics in cancer therapy. Mol Pharm 8(6):2094–2100. 10.1021/mp2002279 21815657 10.1021/mp2002279 PMC3250072

[16] Cunningham JJ, Brown JS, Gatenby RA et al (2018) Optimal control to develop therapeutic strategies for metastatic castrate resistant prostate cancer. J Theor Biol 459:67–78 30243754 10.1016/j.jtbi.2018.09.022

[17] Cunningham JJ, Thuijsman F, Peeters R et al (2020) Optimal control to reach eco-evolutionary stability in metastatic castrate resistant prostate cancer. PLoS ONE 15(12):1–24. 10.1371/journal.pone.0243386 10.1371/journal.pone.0243386 PMC7723267 33290430

[18] Deeks SG, Lewin SR, Havlir DV (2013) The end of AIDS: HIV infection as a chronic disease. The Lancet 382(9903):1525–1533. 10.1016/S0140-6736(13)61809-7 10.1016/S0140-6736(13)61809-7 PMC4058441 24152939

[19] Dimopoulos MA, Jakubowiak AJ, McCarthy PL et al (2020) Developments in continuous therapy and maintenance treatment approaches for patients with newly diagnosed multiple myeloma. Blood Cancer J 10(2):1–19. 10.1038/s41408-020-0273-x 32054831 10.1038/s41408-020-0273-x PMC7018731

[20] Dingli D, Chalub FdC, Santos F et al (2009) Cancer phenotype as the outcome of an evolutionary game between normal and malignant cells. Br J Cancer 101(7):1130–1136 19724279 10.1038/sj.bjc.6605288 PMC2768082

[21] Dong L, Zieren RC, Xue W et al (2019) Metastatic prostate cancer remains incurable, Why? Asian J Urol 6(1):26–41. 10.1016/j.ajur.2018.11.005 30775246 10.1016/j.ajur.2018.11.005 PMC6363601

[22] Dujon AM, Aktipis A, Alix-Panabières C et al (2021) Identifying key questions in the ecology and evolution of cancer. Evol Appl 14(4):877–892. 10.1111/eva.13190 33897809 10.1111/eva.13190 PMC8061275

[23] Enriquez-Navas PM, Kam Y, Das T et al (2016) Exploiting evolutionary principles to prolong tumor control in preclinical models of breast cancer. Sci Transl Med 8(327):327ra24. 10.1126/scitranslmed.aad7842 10.1126/scitranslmed.aad7842 PMC4962860 26912903

[24] Evans CP (2018) Bipolar androgen therapy: an intriguing paradox. Lancet Oncol 19(1):8–10. 10.1016/S1470-2045(17)30907-5 29248235 10.1016/S1470-2045(17)30907-5

[25] Frei E, Elias A, Wheeler C et al (1998) The relationship between high-dose treatment and combination chemotherapy: the concept of summation dose intensity. Clin Cancer Res 4(9):2027–2037 9748116

[26] Freischel AR, Damaghi M, Cunningham JJ et al (2021) Frequency-dependent interactions determine outcome of competition between two breast cancer cell lines. Sci Rep. 10.1038/s41598-021-84406-3 10.1038/s41598-021-84406-3 PMC7921689 33649456

[27] Gad S (2014) Maximum tolerated dose. In: Wexler P (ed) Encyclopedia of toxicology, 3rd edn. Academic Press, Oxford, p 164

[28] Gatenby R (2009) A change of strategy in the war on cancer. Nature 459(7246):508–509. 10.1038/459508a 19478766 10.1038/459508a

[29] Gatenby RA, Brown JS (2020) Integrating evolutionary dynamics into cancer therapy. Nat Rev Clin Oncol 17(11):675–686. 10.1038/s41571-020-0411-1 32699310 10.1038/s41571-020-0411-1

[30] Gatenby RA, Brown J, Vincent T (2009) Lessons from applied ecology: cancer control using an evolutionary double bind. Can Res 69(19):7499–7502. 10.1158/0008-5472.CAN-09-1354 10.1158/0008-5472.CAN-09-1354 19752088

[31] Gatenby RA, Silva AS, Gillies RJ et al (2009) Adaptive therapy. Can Res 69(11):4894–4903. 10.1158/0008-5472.CAN-08-3658 10.1158/0008-5472.CAN-08-3658 PMC3728826 19487300

[32] Gatenby RA, Zhang J, Brown JS (2019) First strike-second strike strategies in metastatic cancer: lessons from the evolutionary dynamics of extinction. Can Res 79(13):3174–3177. 10.1158/0008-5472.CAN-19-0807 10.1158/0008-5472.CAN-19-0807 PMC6606376 31221821

[33] Gatenby RA, Artzy-Randrup Y, Epstein T et al (2020) Eradicating metastatic cancer and the eco-evolutionary dynamics of anthropocene extinctions. Can Res 80(3):613–623. 10.1158/0008-5472.CAN-19-1941 10.1158/0008-5472.CAN-19-1941 PMC7771333 31772037

[34] Ghaffari Laleh N, Loeffler CML, Grajek J et al (2022) Classical mathematical models for prediction of response to chemotherapy and immunotherapy. PLoS Comput Biol 18(2):e1009822. 10.1371/journal.pcbi.1009822 35120124 10.1371/journal.pcbi.1009822 PMC8903251

[35] Gluzman M, Scott JG, Vladimirsky A (2020) Optimizing adaptive cancer therapy: dynamic programming and evolutionary game theory. Proc R Soc B 287(1925):20192454. 10.1098/rspb.2019.2454 10.1098/rspb.2019.2454 PMC7211445 32315588

[36] Greaves M, Maley CC (2012) Clonal evolution in cancer. Nature 481:306–313. 10.1038/nature10762 22258609 10.1038/nature10762 PMC3367003

[37] Henley SJ, Ward EM, Scott S et al (2020) Annual report to the nation on the status of cancer, part I: national cancer statistics. Cancer 126(10):2225–2249. 10.1002/cncr.32802 32162336 10.1002/cncr.32802 PMC7299151

[38] Hicks JR, von Stackelberg H (1935) Marktform und Gleichgewicht. Econ J 45(178):334. 10.2307/2224643 (https://www.jstor.org/stable/10.2307/2224643?origin=crossref)

[39] Hummert S, Bohl K, Basanta D et al (2014) Evolutionary game theory: cells as players. Mol BioSyst 10(12):3044–3065 25270362 10.1039/c3mb70602h

[40] Iannelli M, Pugliese A (2014) An introduction to mathematical population dynamics, UNITEXT, vol 79. Springer, Cham. 10.1007/978-3-319-03026-5

[41] Jalali R, Dutta D (2012) Factors influencing quality of life in adult patients with primary brain tumors. Neuro-oncology 14(suppl-4):iv8–iv16 23095834 10.1093/neuonc/nos205 PMC3480247

[42] Jemal A, Ward EM, Johnson CJ et al (2017) Fibroblasts and alectinib switch the evolutionary games played by non-small cell lung cancer 1975-2014, featuring survival. JNCI: J Natl Cancer Inst 109(9):djx030. 10.1093/jnci/djx030 28376154 10.1093/jnci/djx030 PMC5409140

[43] Kaznatcheev A, Vander Velde R, Scott JG et al (2017) Cancer treatment scheduling and dynamic heterogeneity in social dilemmas of tumour acidity and vasculature. Br J Cancer 116:785–792. 10.1038/bjc.2017.5 28183139 10.1038/bjc.2017.5 PMC5355932

[44] Kaznatcheev A, Peacock J, Basanta D et al (2019) Fibroblasts and alectinib switch the evolutionary games played by non-small cell lung cancer. Nature Ecol Evolut 3(3):450–456. 10.1038/s41559-018-0768-z 10.1038/s41559-018-0768-z PMC6467526 30778184

[45] Kleshnina M, Streipert S, Brown JS et al (2023) Game theory for managing evolving systems: challenges and opportunities of including vector-valued strategies and life-history traits. Dyn Games Appl 13(4):1130–1155. 10.1007/s13235-023-00544-5 (https://link.springer.com/10.1007/s13235-023-00544-5)

[46] Kuang Y, Nagy JD, Eikenberry SE (2016) Introduction to mathematical oncology. Chapman and Hall/CRC, London

[47] Liberti MV, Locasale JW (2016) The Warburg effect: How does it benefit cancer cells? Trends Biochem Sci 41(3):211–218. 10.1016/j.tibs.2015.12.001 (https://linkinghub.elsevier.com/retrieve/pii/S0968000415002418) 26778478 10.1016/j.tibs.2015.12.001 PMC4783224

[48] Lin-Rahardja K, Weaver DT, Scarborough JA et al (2023) Evolution-informed strategies for combating drug resistance in cancer. Int J Mol Sci 24(7):6738 37047714 10.3390/ijms24076738 PMC10095117

[49] Mahungu TW, Rodger AJ, Johnson MA (2009) HIV as a chronic disease. Clin Med 9(2):125. 10.7861/clinmedicine.9-2-125 10.7861/clinmedicine.9-2-125 PMC4952661 19435115

[50] Maley CC, Reid BJ, Forrest S (2004) Cancer prevention strategies that address the evolutionary dynamics of neoplastic cells: simulating benign cell boosters and selection for chemosensitivity. Cancer Epidemiol Prevention Biomark 13(8):1375–1384. 10.1158/1055-9965.1375.13.8 15298961

[51] Masud M, Kim E (2024) Theoretical understanding of evolutionary dosing following tumor dynamics. Chaos Solitons Fractals 179:114451

[52] Merker VL, Bredella MA, Cai W et al (2014) Relationship between whole-body tumor burden, clinical phenotype, and quality of life in patients with neurofibromatosis. Am J Med Genet A 164(6):1431–1437 10.1002/ajmg.a.36466 24664633

[53] Merlo LM, Pepper JW, Reid BJ et al (2006) Cancer as an evolutionary and ecological process. Nat Rev Cancer 6(12):924–935. 10.1038/nrc2013 17109012 10.1038/nrc2013

[54] Mitola G, Falvo P, Bertolini F (2021) New insight to overcome tumor resistance: an overview from cellular to clinical therapies. Life 11(11):1131 34833007 10.3390/life11111131 PMC8621237

[55] Muros FJ, Maestre JM, You L, et al (2017) Model predictive control for optimal treatment in a spatial cancer game. In: 2017 IEEE 56th annual conference on decision and control (CDC), pp 5539–5544, 10.1109/CDC.2017.8264481

[56] Natterson-Horowitz B, Aktipis A, Fox M et al (2023) The future of evolutionary medicine: sparking innovation in biomedicine and public health. Front Sci 1:997136 37869257 10.3389/fsci.2023.997136 PMC10590274

[57] van Neerven SM, de Groot NE, Nijman LE et al (2021) Apc-mutant cells act as supercompetitors in intestinal tumour initiation. Nature 594(7863):436–441. 10.1038/s41586-021-03558-4 34079128 10.1038/s41586-021-03558-4

[58] Nichol D, Rutter J, Bryant C et al (2019) Antibiotic collateral sensitivity is contingent on the repeatability of evolution. Nat Commun 10(1):334. 10.1038/s41467-018-08098-6 30659188 10.1038/s41467-018-08098-6 PMC6338734

[59] Noble RJ, Walther V, Roumestand C et al (2021) Paracrine behaviors arbitrate parasite-like interactions between tumor subclones. Front Ecol Evol 9:675638. 10.3389/fevo.2021.675638 35096847 10.3389/fevo.2021.675638 PMC8794381

[60] O’Sullivan B, Brierley JD, D’Cruz A et al (2015) UICC manual of clinical oncology. Wiley, Hoboken

[61] Pressley M, Salvioli M, Lewis DB et al (2021) Evolutionary dynamics of treatment-induced resistance in cancer informs understanding of rapid evolution in natural systems. Front Ecol Evol 9:682121. 10.3389/fevo.2021.681121

[62] Rajkumar SV (2011) Treatment of multiple myeloma. Nat Rev Clin Oncol 8(8):479–491. 10.1038/nrclinonc.2011.63 21522124 10.1038/nrclinonc.2011.63 PMC3773461

[63] Rajkumar SV, Kumar S (2020) Multiple myeloma current treatment algorithms. Blood Cancer J 10(9):1–10. 10.1038/s41408-020-00359-2 32989217 10.1038/s41408-020-00359-2 PMC7523011

[64] Reed DR, Metts J, Pressley M et al (2020) An evolutionary framework for treating pediatric sarcomas. Cancer 126(11):2577–2587. 10.1002/cncr.32777 32176331 10.1002/cncr.32777 PMC7318114

[65] Reynolds A (2009) Patient-centered care. Radiol Technol 81(2):133–147 19901351

[66] Salvioli M, Dubbeldam J, Staňková K et al (2021) Fisheries management as a Stackelberg evolutionary game: finding an evolutionarily enlightened strategy. PLoS ONE 16(1):e0245255. 10.1371/journal.pone.0245255 33471815 10.1371/journal.pone.0245255 PMC7817040

[67] Salvioli M, Vandelaer L, Baena E et al (2024) The effect of tumor composition on the success of adaptive therapy: The case of metastatic castrate-resistant prostate cancer. PLoS ONE 19(9):1–15. 10.1371/journal.pone.0308173 10.1371/journal.pone.0308173 PMC11426540 39325718

[68] Savage P, Stebbing J, Bower M et al (2009) Why does cytotoxic chemotherapy cure only some cancers? Nat Clin Pract Oncol 6(1):43–52. 10.1038/ncponc1260 18982000 10.1038/ncponc1260

[69] Simaan M, Cruz JB Jr (1973) On the Stackelberg strategy in nonzero-sum games. J Optim Theory Appl 11(5):533–555. 10.1007/BF00935665

[70] Sledge GW Jr (2016) Curing metastatic breast cancer. J Oncol Pract 12(1):6–10. 10.1200/JOP.2015.008953 26759458 10.1200/JOP.2015.008953

[71] Soboleva A, Kaznatcheev A, Cavill R, et al (2024) Validation of polymorphic Gompertzian model of cancer through in vitro and in vivo data. PLOS ONE, in print 10.1371/journal.pone.0310844 PMC11717199 39787141

[72] Spekking L, Lohk C, Jung W et al (2024) How to use transcriptomic data for game-theoretic modeling of treatment-induced resistance in cancer cells? A case study in patient-derived glioblastoma organoids. bioRxiv. 10.1101/2022.01.26.477755

[73] von Stackelberg H (1934) Marktform und Gleichgewicht. Verlag von Julius Springer, Wien und Berlin

[74] Staňková K (2019) Resistance games. Nat Ecol Evolut 3(3):336–337. 10.1038/s41559-018-0785-y 10.1038/s41559-018-0785-y 30778182

[75] Staňková K, Brown JS, Dalton WS et al (2019) Optimizing cancer treatment using game theory. JAMA Oncol 5(1):96–103. 10.1001/jamaoncol.2018.3395 30098166 10.1001/jamaoncol.2018.3395 PMC6947530

[76] Stein A, Salvioli M, Garjani H et al (2023) Stackelberg evolutionary game theory: How to manage evolving systems. Philos Trans R Soc B 378(1876):20210495. 10.1098/rstb.2021.0495 10.1098/rstb.2021.0495 PMC10024980 36934755

[77] Strobl MA, West J, Viossat Y et al (2021) Turnover modulates the need for a cost of resistance in adaptive therapy. Can Res 81(4):1135–1147 10.1158/0008-5472.CAN-20-0806 PMC8455086 33172930

[78] Strobl MA, Martin AL, West J et al (2024) To modulate or to skip: De-escalating parp inhibitor maintenance therapy in ovarian cancer using adaptive therapy. Cell Syst 15(6):510-525.e6. 10.1016/j.cels.2024.04.003 38772367 10.1016/j.cels.2024.04.003 PMC11190943

[79] Susswein Z, Sengupta S, Clarke R, et al (2022) Borrowing ecological theory to infer interactions between sensitive and resistant breast cancer cell populations. bioRxiv 10.1101/2022.02.18.481041

[80] Takimoto CH (2009) Maximum tolerated dose: Clinical endpoint for a bygone era? Target Oncol 4(2):143–147. 10.1007/s11523-009-0108-y 19377851 10.1007/s11523-009-0108-y

[81] Tomlinson IP (1997) Game-theory models of interactions between tumour cells. Eur J Cancer 33(9):1495–1500 9337695 10.1016/s0959-8049(97)00170-6

[82] Vasan N, Baselga J, Hyman DM (2019) A view on drug resistance in cancer. Nature 575:299–309. 10.1038/s41586-019-1730-1 31723286 10.1038/s41586-019-1730-1 PMC8008476

[83] Vincent TL, Brown JS (2005) Evolutionary game theory, natural selection, and Darwinian dynamics. Cambridge University Press, Cambridge

[84] Viossat Y, Noble R (2021) A theoretical analysis of tumour containment. Nat Ecol Evolut 5(6):826–835. 10.1038/s41559-021-01428-w 10.1038/s41559-021-01428-w PMC8967123 33846605

[85] Werner B, Lutz D, Brümmendorf TH et al (2011) Dynamics of resistance development to imatinib under increasing selection pressure: a combination of mathematical models and in vitro data. PLoS ONE 6(12):e28955. 10.1371/journal.pone.0028955 22216147 10.1371/journal.pone.0028955 PMC3245228

[86] West J, Adler F, Gallaher J et al (2023) A survey of open questions in adaptive therapy: bridging mathematics and clinical translation. eLife 12:e84263. 10.7554/eLife.84263 36952376 10.7554/eLife.84263 PMC10036119

[87] Wilson M, Weinreb J, Hoo GWS (2007) Intensive insulin therapy in critical care. Diabetes Care 30(4):1005–1011. 10.2337/dc06-1964 17213376 10.2337/dc06-1964

[88] Wölfl B, te Rietmole H, Salvioli M et al (2021) The contribution of evolutionary game theory to understanding and treating cancer. Dyn Games Appl. 10.1007/s13235-021-00397-w 10.1007/s13235-021-00397-w PMC9117378 35601872

[89] Yoon N, Vander Velde R, Marusyk A et al (2018) Optimal therapy scheduling based on a pair of collaterally sensitive drugs. Bull Math Biol 80(7):1776–1809. 10.1007/s11538-018-0434-2 29736596 10.1007/s11538-018-0434-2

[90] Yoon N, Krishnan N, Scott J (2021) Theoretical modeling of collaterally sensitive drug cycles: shaping heterogeneity to allow adaptive therapy. J Math Biol 83(5):1–29. 10.1007/s00285-021-01671-6 34632539 10.1007/s00285-021-01671-6

[91] You L, Brown J, Thuijsman F et al (2017) Spatial vs. non-spatial eco-evolutionary dynamics in a tumor growth model. J Theor Biol 435:78–97. 10.1016/j.jtbi.2017.08.022. (**Epub 2017 Sep 21 PMID: 28870617**) 28870617 10.1016/j.jtbi.2017.08.022

[92] Zhang J, Cunningham J, Brown JS et al (2017) Integrating evolutionary dynamics into treatment of metastatic castrate-resistant prostate cancer. Nat Commun 8(1):1816. 10.1038/s41467-017-01968-5 29180633 10.1038/s41467-017-01968-5 PMC5703947

[93] Zhang J, Cunningham J, Brown JS et al (2022) Evolution-based mathematical models significantly prolong response to abiraterone in metastatic castrate-resistant prostate cancer and identify strategies to further improve outcomes. eLife 11:e76284. 10.7554/eLife.76284 35762577 10.7554/eLife.76284 PMC9239688

